# Investigations of the tick burden on passeriform, water-associated and predatory birds reveal new tick–host associations and habitat-related factors of tick infestation

**DOI:** 10.1186/s13071-024-06229-1

**Published:** 2024-03-18

**Authors:** Andor Pitó, Boglárka Bukor, Előd Győrig, Vojtěch Brlík, Jenő Kontschán, Gergő Keve, Nóra Takács, Sándor Hornok

**Affiliations:** 1 Department of Parasitology and Zoology, University of Veterinary Medicine, Budapest, Hungary; 2 BirdLife Hungary, Budapest, Hungary; 3 HUN-REN-PE Evolutionary Ecology Research Group, University of Pannonia, Pf. 1158, 8210 Veszprém, Hungary; 4 Behavioral Ecology Research Group, Center for Natural Sciences, University of Pannonia, Veszprém, Hungary; 5 Department of Ecology, Charles University, Prague, Czechia; 6 Institute of Vertebrate Biology, Czech Academy of Sciences, Brno, Czechia; 7 Plant Protection Institute, HUN-REN Centre for Agricultural Research, Budapest, Hungary; 8 Department of Plant Sciences, Albert Kázmér Faculty of Mosonmagyaróvár, Széchenyi István University, Mosonmagyaróvár, Hungary; 9 HUN-REN-UVMB Climate Change: New Blood-Sucking Parasites and Vector-Borne Pathogens Research Group, Budapest, Hungary

**Keywords:** Ixodidae, Passeriformes, Accipitriformes, Pelecaniformes

## Abstract

**Background:**

Previous studies on the tick infestation of birds in the Carpathian Basin focused on songbirds (Passeriformes). Thus, the primary aim of the present work was to extend the scope of previous studies, i.e. to include aquatic (water-associated) bird species in a similar context, especially considering that these birds are usually long-distance migrants.

**Methods:**

Between March 2021 and August 2023, 11,919 birds representing 126 species were checked for the presence of ticks. From 352 birds belonging to 40 species, 905 ixodid ticks were collected. Tick species were identified morphologically and/or molecularly.

**Results:**

Ticks from avian hosts belonged to seven species: *Ixodes ricinus* (*n* = 448), *I. frontalis* (*n* = 31), *I. festai* (*n* = 2), *I. arboricola* (*n* = 36), *I. lividus* (*n* = 4), *Haemaphysalis concinna* (*n* = 382) and *Dermacentor reticulatus* (*n* = 2). Nymphs of *I. ricinus* occurred with a single activity peak around March–May, whereas its larvae typically infested birds in May, June or July. By contrast, *H. concinna* usually had its activity maximum during the summer (nymphs in June–July, larvae later in July–August). Interestingly, two ornithophilic species, *I. frontalis* and *I. arboricola*, were most active around winter months (between October and April). A significantly lower ratio of aquatic birds was found tick-infested than songbirds. Several new tick–host associations were revealed, including *I. ricinus* from Greylag Goose (*Anser anser*) and *D. reticulatus* from Great Egret (*Ardea alba*) and Sedge Warbler (*Acrocephalus schoenobaenus*). Ticks were collected for the first time in Europe from two species of predatory birds as well as from Little Bittern (*Ixobrychus minutus*). Bird species typically inhabiting reedbeds were most frequently infested with *H. concinna*, and most ticks localized at their throat, as opposed to forest-dwelling avian hosts, on which *I. ricinus* predominated and ticks were more evenly distributed.

**Conclusions:**

In the evaluated region, aquatic birds appear to be less important in tick dispersal than songbirds. However, newly revealed tick-host associations in this category attest to their hitherto neglected contribution. The results suggest that the habitat type will have significant impact not only on the species composition but also on the feeding location of ticks on birds.

**Graphical Abstract:**

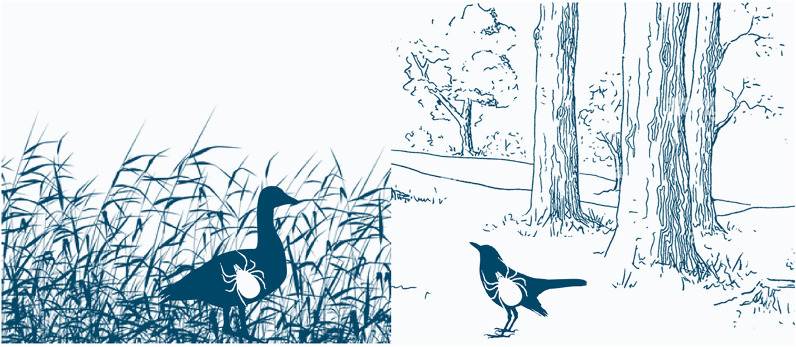

**Supplementary Information:**

The online version contains supplementary material available at 10.1186/s13071-024-06229-1.

## Background

Hard ticks (Acari: Ixodidae) affect animal and human life in several ways, among which the most important is that they are transmitters (vectors) of a broad range of pathogens with high veterinary-medical significance [1]. Because each species of tick-borne pathogens has evolved to associate with certain tick vectors in which, as biological vectors, they can multiply and can reach the form infectious for susceptible vertebrate hosts, the local tick fauna will determine the endemic occurrence of tick-borne diseases [2]. In this context, the highest contribution to local tick populations is probably achieved by birds, in terms of introducing new individuals and even new species [3]. Their role in tick dispersal in urban areas is particularly high [4], especially when considering that in cities they outnumber medium to large mammals. Furthermore, birds are important carriers and distributors of ticks and thus of tick-borne pathogens, even on a transcontinental scale [5].

The history of investigating the tick infestation of birds in Central Europe and the Carpathian Basin goes back several decades [6]. Most of these studies in the past were either based on random, sporadic tick collections [7] or annual removal of ticks from birds at one stop-over site [8, 9]. In 2022, however, a country-wide survey on ticks associated with avian hosts was also conducted [10]. Nevertheless, all these bird tick studies focused on just one order of birds, the Passeriformes (songbirds).

Aquatic birds, for instance members of the order Anseriformes (ducks, geese, swans), are not known as hosts of ticks in Hungary [11] and were also rarely reported in this role in Europe [12]. One of the underlying reason why anseriform birds appear to be neglected when studying avian tick hosts is probably the difficulty of sampling, i.e. they cannot be easily mist-netted and are rather funnel trapped or caught with other methods. At the same time, aquatic birds in general were reported to develop seroconversion to tick-borne encephalitis virus (TBEV) in Europe [13], and ducks in particular have been recently shown to develop viremia to this pathogen [14], justifying their epidemiological role. In addition, while geese are presumably main tick hosts and main vertebrate *Borrelia* reservoirs [15] and are long-known natural carriers of *Ixodes ricinus*-borne pathogens [16], during the past decades one of their most widespread species, the Greylag Goose (*Anser anser*) was not reported with tick infestation in Europe [12].

Thus, the aim of this study was to extend the scope of previous surveys on the role of birds as tick hosts in the Carpathian Basin, i.e. to investigate the following new aspects: (i) regions and habitat types previously not evaluated; (ii) avian hosts species which are rare or for other reasons were previously not examined; (iii) data on the anatomical location of tick infestation hitherto not analyzed in the context of avian traits influencing it; (iv) the tick burden of aquatic (water-associated) bird species, especially considering that these birds are usually long-distance migrants.

## Methods

### Sample collection and identification of tick species

**Fig. 1:**
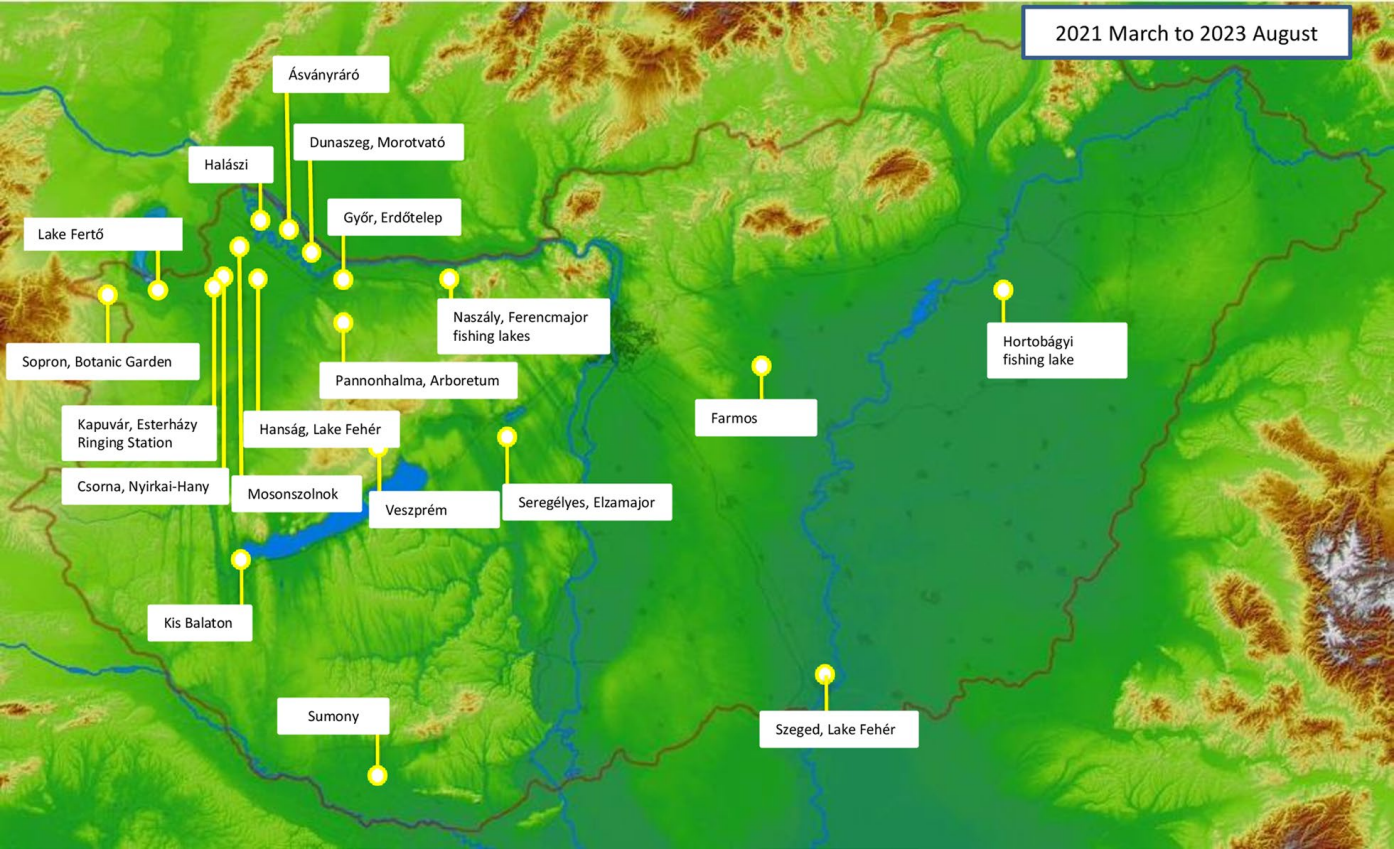
Map of Hungary showing the collection sites

During this study, 11,919 birds representing 126 species were checked for the presence of ticks. The samples were collected during bird ringing activities between March 2021 and August 2023 at 19 locations focusing on northwestern Hungary (Fig. 1). Songbirds (order Passeriformes) were captured with standard mist nets (Ecotone, Gdansk, Poland), which are 12 m long and 2.5 m high. Depending on the habitat, 1–12 nets were used. For water-associated birds from other orders (Anseriformes, Pelecaniformes, Gruiformes, Charadriiformes, Podicipediformes, Ciconiiformes), occasionally manual capture was also applied. In addition, rescued predatory birds (orders Accipitriformes, Falconiformes) were also included in the study. All birds were carefully inspected for the presence of ticks, starting from the throat and progressing by blowing the plumage through the beak, eyes and then ears. Ticks were removed from the birds with pointed tweezers and placed into pre-numbered tubes filled with 96% ethanol. Tick species were identified according to standard keys [17]. In addition, the species identity of *Dermacentor reticulatus* nymph was confirmed molecularly based on the 16S rRNA gene (data not shown), as reported [10].

### Statistical analysis

Prevalence data were compared with Fisher's exact test (https://www.langsrud.com/fisher.htm), and differences were considered significant if *P* < 0.05. Bird species were assigned into categories according to their typical habitat (Table 2; Additional file 1) as previously reported [10].

## Results

### Species and developmental stages of ticks collected from birds

**Fig. 2:**
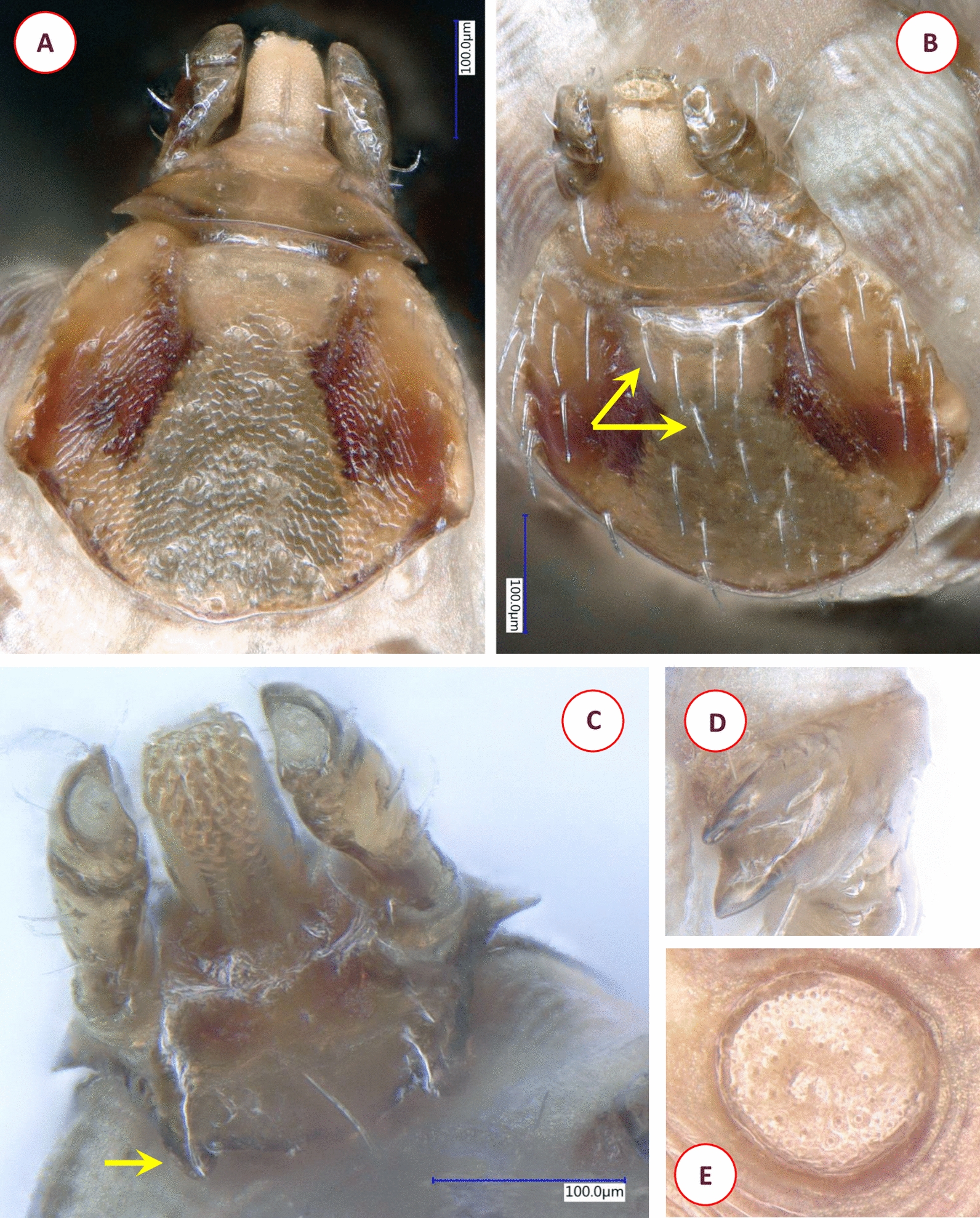
Morphology of the *Dermacentor reticulatus* nymph collected from Sedge Warbler (*Acrocephalus schoenobaenus*) in Hungary: (**A**) scutum and basis capituli, dorsal view; (**B**) scutum and basis capituli, anterior view showing long (50 μm) scutal setae (arrows); (**C**) basis capituli, ventral view (arrow: prominent auricula); (**D**) divided coxa I; (**E**) rounded spiracular plate

A total of 905 ticks belonging to the following seven species were collected from birds: *I. ricinus* (*n* = 448: 173 larvae, 275 nymphs); *I. frontalis* (*n* = 31: 15 larvae, 11 nymphs, five females); *I. festai* (*n* = 2: two females); *I. arboricola* (*n* = 36: 21 larvae, 14 nymphs, one female); *I. lividus* (*n* = 4: four females); *Haemaphysalis concinna* (*n* = 382: 145 larvae, 237 nymphs); *D. reticulatus* (*n* = 2: one nymph (Fig. 2) and one female).

### Seasonal distribution of ticks collected from birds

Due to the random distribution of sample collection days (Additional file 2), only larger monthly or seasonal trends were followed. Considering the two most frequently collected species, the occurrence maxima of the different stages are shown in Table 1. Thus, in the case of *I. ricinus* nymphs, there was typically a spring, March-to-May peak in their activity. The occurrence of larvae mostly showed a peak between May and July, which was also different between the years. The second most common species was *H. concinna*, for which the presence on birds in spring and autumn periods is seldom observed in Hungary (Table 1). The occurrence of nymphs of this species reached its maximum in June–July of each year; then, in July–August (typically by mid-August), this was taken over by the predominance of larvae.

**Table 1:** Seasonal distribution of ticks and their developmental stages/sex, collected from birds in the study period (March 2021 to August 2023)

	*Ixodes ricinus*	*Ixodes frontalis*	*Ixodes festai*	*Ixodes arboricola*	*Ixodes lividus*	*Haemaphysalis concinna*	*Dermacentor reticulatus*
	Larva/nymph/female	Larva/nymph/female	Larva/nymph/female	Larva/nymph/female	Larva/nymph/female	Larva/nymph/female	Larva/nymph/female
2021 March	–	–	–	–	–	–	–
2021 April	0/4/0	–	0/0/1	–	–	–	–
2021 May	–	–	–	–	–	–	–
2021 June	–	–	–	–	–	–	0/0/1
2021 July	1/5/0	–	–	–	–	1/16/0	0/1/0
2021 August	4/1/0	–	–	–	–	0/2/0	–
2021 September	3/0/0	–	–	–	–	0/1/0	–
2021 October	0/1/0	–	–	–	–	–	–
2021 November	–	1/0/0	–	–	–	–	–
2021 December	–	–	–	–	–	–	–
2022 January	–	–	–	–	–	–	–
2022 February	–	–	–	–	–	–	–
2022 March	–	–	–	–	–	–	–
2022 April	0/15/0	–	–	–	–	0/1/0	–
2022 May	34/57/0	–	–	–	–	–	–
2022 June	4/3/0	–	–	–	0/0/2	2/24/0	–
2022 July	6/6/0	–	–	–	-	5/44/0	–
2022 August	9/14/0	0/1/0	–	–	-	16/8/0	–
2022 September	1/1/0	–	–	–	-	2/2/0	–
2022 October	–	0/1/0	–	–	–	–	–
2022 November	–	–	–	–	–	–	–
2022 December	0/11/0	11/2/1	–	21/14/1	–	–	–
2023 January	–	0/1/1	–	–	–	–	–
2023 February	–	–	–	–	–	–	–
2023 March	15/88/0	3/5/2	0/0/1	–	–	1/1/0	–
2023 April	2/32/0	0/2/0	–	–	–	0/4/0	–
2023 May	–	–	–	–	–	-	–
2023 June	0/3/0	–	–	–	0/0/2	9/30/0	–
2023 July	85/17/0	–	–	–	–	85/98/0	–
2023 August	7/11/0	–	–	–	–	24/6/0	–

The third most common species was *I. frontalis*, which occurred between October and April: larvae had their peak activity between October and December while nymphs between January and April (Table 1). As an exception, a single nymph was found on a bird in August. The occurrence of females appeared to be random. *Ixodes arboricola* is the fourth species that was collected in larger numbers. Findings of this species were restricted to one place (Fig. 1: Halászi), where it was the most common tick species on birds on two ringing days in December. It was found from a total of 11 birds (*n* = 36), including larvae (*n* = 21), nymphs (*n* = 14) and females (*n* = 1). By contrast, in the relevant period, *I. ricinus* occurred on 10 birds (*n* = 12) and *I. frontalis* was collected from nine (*n* = 14).

### Host associations of ticks collected from birds

The distributions of tick species and stages according to host species and bird orders are shown in Table 2. During the investigation, 352 individuals of 40 bird species were found to be infested with ticks. Water-associated avian hosts examined in this study (*n* = 1497) belonged to 53 species (data not shown) and six orders (see above). Among these, only five tick-infested individuals of three species were found (Table 2). This is significantly (*P* < 0.0001) fewer than in the case of songbirds (Passeriformes), among which 10,422 birds of 73 species were examined, and 340 individuals representing 32 species were tick-infested (Table 2). Ticks were also collected from four birds of prey (Table 2).

**Table 2:** Summary of tick species and developmental stages according to avian host taxa

Hose			*Ixodes ricinus*			*Ixodes frontalis*			*Haemaphysalis concinna*			Other tick species		
Order	Sp. code	*n* =	L	N	F	L	N	F	L	N	F	L	N	F
Passeriformes	LANCOL^2^	1		1					4					
	PARCAE	2										Ia(6)	Ia(6)	
	PARMAJ^2^	57	92	77		11	2	1	1	7		Ia(15)	Ia(8)	Ia(1)
	PANBIA^1^	6	1	7										
	RIRRIP	4												Il(4)
	AEGCAU	1		3										
	PHYCOL	2		1			1							
	ACRARU^1^	16	5	5					13	10				
	ACRMEL^1^	8	2	1						5				
	ACRSCH^1^	43	3	9				1	22	42			Dr(1)	
	ACRSCI^1^	50	13	25			3		14	22				
	ACRRIS^1^	18	4	9					3	14				
	LOCLUS^1^	50		14					62	97				If(1)
	LOCNAE	1							14					
	SYLATR^2^	7	6	1					1	2				
	SYLCOM^2^	2	1	1										
	REGIGN	1		1										
	CERFAM	1	1											
	CERBRA	1	1											
	STUVUL	2		5										
	TURMER^2^	16	20	37		3			1	5				
	TURPIL	1						1						
	TURPHI	2		5						1				
	ERIRUB^2^	9	5	3		1	4							
	LUSSVE^1^	1	1											
	LUSLUS	1		1										
	LUSMEG^2^	9	7	4					6	5				
	PASMON	3	1	1			1							
	PRUMOD^2^	17	8	42				1	1					If(1)
	COCCOC	2		2										
	CARCHL	2	1	5										
	EMBSCH^1^	6						1	1	5				
Falconiformes	FALTIN	1								1				
Accipitriformes	PERAPI	1								1				
	AQUHEL	1								3				
	CIRAER	1		2					2	15				
Pelecaniformes	IXOMIN	1								1				
	EGRALB	1												Dr(1)
Anseriformes	ANSANS^1^	3	1	13										
Galliformes	COTCOT	1								1				
Total	40	352	173	275		15	11	5	145	237		Ia(21)	Ia(14); Dr(1)	Ia(1); If(2); Il(4); Dr(1)

The third column (*n* =) shows the numbers of individuals of a bird species that were found tick-infested. Species codes (Additional file 1) of birds typically occurring in reedbed habitats are shown with superscript “1” and those with forest habitats with superscript “2”

*L* larva, *N* nymph, *F* female, *sp.* species, *Ia* *Ixodes arboricola*, *If* *Ixodes festai*, *Il* *Ixodes lividus*, *Dr* *Dermacentor reticulatus*

Among songbirds, the most common host of ticks (from which 215 specimens of four tick species were collected) was the Great Tit (*Parus major*) (*n* = 57), followed by the Savi's Warbler (*Locustella luscinioides*) (*n* = 50, with 174 ticks) and then the Eurasian Reed Warbler (*Acrocephalus scirpaceus*) (*n* = 50, with 77 ticks) and the Sedge Warbler (*A. schoenobaenus*) (*n* = 43, with 78 ticks). Less common hosts of *I. ricinus* (carrying a single tick) included the Common Firecrest (*Regulus ignicapillus*), Eurasian Treecreeper (*Certhia familiaris*), Short-toed Treecreeper (*Certhia brachydactyla*) and the Bluethroat (*Luscinia svecica*) (Table 2). Considering the rare hosts of *I. frontalis*, it was removed from a Fieldfare (*Turdus pilaris*), a Common Reed Bunting (*Emberiza schoeniclus*) and a Eurasian Tree Sparrow (*Passer montanus*) (Table 2). *Ixodes festai* was collected twice in the spring during the 3-year study, in both cases females from Savi’s Warbler (*L. luscinioides*) and Dunnock (*Prunella modularis*).

Among the birds of prey (orders Falconiformes, Accipitriformes), tick infestation was observed in four species (Table 2). The highest number, a total of 19 ticks, were collected from the Western Marsh Harrier (*Circus aeruginosus*), of which two *I. ricinus* specimens were identified along with two *H. concinna* larvae and 15 nymphs. Of the remaining species (Common Kestrel: *Falco tinnunculus*, European Honey Buzzard: *Pernis apivorus*, Western Marsh Harrier: *Circus aeruginosus*, Eastern Imperial Eagle: *Aquila heliaca*), only nymphs of *H. concinna* were collected.

Among the birds associated with wetlands, tick infestation was detected among Greylag Goose (*A. anser*) chicks. Ticks were found on 37.5% of chicks (*n* = 3) < 2 weeks old, all of which belonged to *I. ricinus* (1 larva, 13 nymphs). *Dermacentor reticulatus* specimens were collected from birds on two occasions: for the first time in June (2021) a female tick from the tibiotarso-tarsometatarsal joint of a Great Egret (*A. alba*) chick in the nest and for the second time in July (2021) a nymph was removed from a Sedge Warbler (*A. schoenobaenus*) near Lake Fehér (Fig. 2). In addition, we found the species *H. concinna* on Little Bittern (*Ixobrychus minutus*) and among galliform birds on Common Quail (*Coturnix coturnix*).

### Species of ticks according to the feeding habitat of their avian hosts

Tick-infested bird species were also compared according to their typical habitat. Among bird species that are reed-associated (Table 2), infestation with *H. concinna* dominated (*n* = 293 *H. concinna* vs. *n* = 77 *I. ricinus*), while for members of the forest ecosystem (Table 2), infestation with *I. ricinus* was more typical (*n* = 29 *H. concinna* vs. *n* = 302 *I. ricinus*). This was a highly significant (*P* < 0.0001) difference.

### Anatomic location of ticks on their avian hosts

**Fig. 3:**
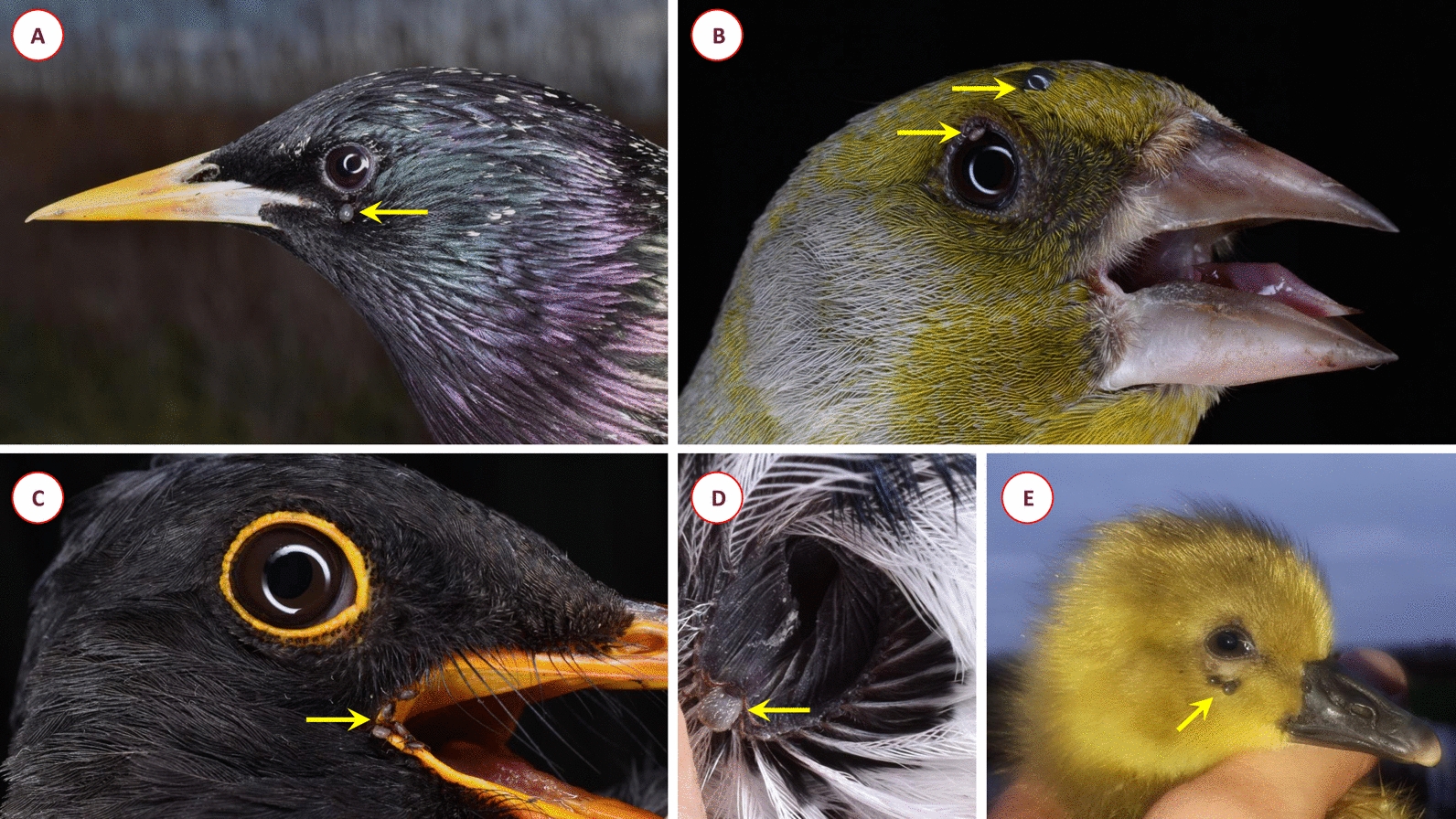
Anatomical location of tick infestation among birds in this study: (**A**) below the eye in Common Starling (*Sturnus vulgaris*); (**B**) on the eyelid and on the vertex in European Greenfinch (*Chloris chloris*); (**C**) in the corner of beaks, margin of oral mucosa in Eurasian Blackbird (*Turdus merula*); (**D**) in the *meatus auditorius* in Eurasian Blue Tit (*Cyanistes caeruleus*); (**E**) below the eyes in Greylag Goose (*Anser anser*)

The anatomical location occupied by ticks during blood sucking was examined for 100 birds, from which a total of 122 ticks were removed. Individuals of 18 bird species were included in this analysis. Most ticks were found in the throat region (*n* = 73), but they also occurred around the eyes (*n* = 15), in the corner of the beak (*n* = 24), in the ear canal (*n* = 6) and rarely in unusual places such as around the cloaca (*n* = 1), on the wing (*n* = 2) and on the top of the head (*n* = 1) (Fig. 3). Most ticks were collected from Savi’s Warbler (*L. luscinioides*), in which we found ticks around the throat in all cases (*n* = 19). In the case of the Great Tit (*P. major*) and Common Nightingale (*Luscinia megarhynchos*), ticks occurred with the same frequency around the corner of the beak and the eyes as in the areas around the throat.

**Fig. 4:**
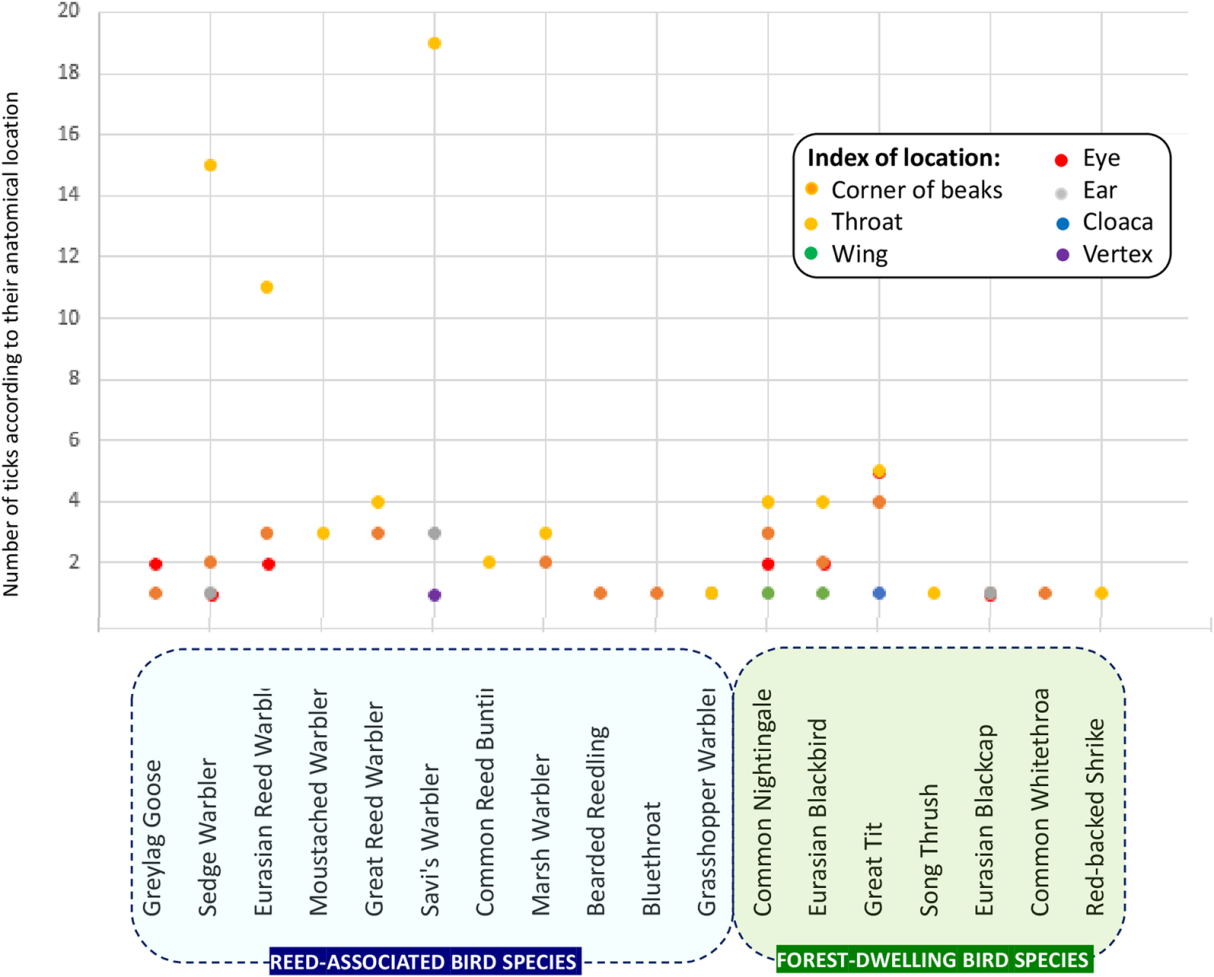
Number of ticks removed from 100 birds according to the anatomical location of tick infestation, species of avian hosts and their typical habitat

Interestingly, the typical tick predilection sites differed according to reed-associated or forest-dwelling bird species. Among bird species common in reedbeds (Fig. 4), tick infestation of the throat area dominated (*n* = 54 ticks in the throat area vs. *n* = 16 ticks in other places), while for forest-dwelling bird species (Fig. 4), the localization was significantly (*P* = 0.0003) less frequent around the throat and was more evenly distributed (*n* = 13 throat area, *n* = 10 eye, *n* = 10 beak corner).

## Discussion

During the 3 years of the study, mostly preadult developmental stages of seven tick species were collected from birds. This means that, with the exception of four species (two *Hyalomma* species, *Haemaphysalis punctata* and *Dermacentor marginatus*), all tick species reported so far from birds in Hungary were found [12].

The seasonality of different tick species on birds matched previous Central European results [7, 18–21]. Accordingly, the spring peak of *I. ricinus* nymphs varied between the months of March and May during the 3 years of the study. The larvae reached their maximum level of infestation between May and July. During this study, *I. ricinus* was collected from several bird species, from which tick infestations had not been previously reported in Hungary or Europe [12]. Thus, new hosts of this tick species in Hungary include the Common Firecrest (*R. ignicapillus*) and Eurasian Treecreeper (*C. familiaris*). In addition, *I. ricinus* was also found on a Greylag Goose (*A. anser*). This result is especially important not only because tick infestation of this bird species has not been previously reported in Europe [12], but anseriform birds are also long-known to play a role in the epidemiological cycle of *I. ricinus*-borne pathogens, including the tick-borne encephalitis virus [13, 14] and borreliae [15].

The second most common tick species was *H. concinna*. The number of nymphs reached its maximum in June–July of each year, and then gradually the larvae became more prevalent on birds in July–August. According to previous studies, the larvae are active from the end of May to October, while the nymphs occur from April to October, but as in the present survey, they were most common in the middle and end of summer [18, 22].

Most bird species that were found infested with *H. concinna* in this study are known to be associated with reedbeds, similarly to the results of a long-term study on bird-associated ticks in Hungary [23]. These hosts were already reported [7]. In addition, for the first time in Europe, we have recorded *H. concinna* infestation on Common Kestrel (*F. tinnunculus*), European Honey Buzzard (*P. apivorus*), Western Marsh Harrier (*C. aeruginosus*), Eastern Imperial Eagle (*A. heliaca*) and Little Bittern (*I. minutus*) [12]. Moreover, no ticks from the Western Marsh Harrier (*C. aeruginosus*), the Eastern Imperial Eagle (*A. heliaca*) and the Little Bittern (*I. minutus*) have ever been found in Europe [12].

The third most common species was *I. frontalis*, which almost exclusively occurred between October and April. This is in line with previous results when peak activity was observed between January and April, with rare collection days in August [7], similarly to this study. Larvae were more common between October and December while nymphs between March and April. The occurrence of adults showed a random distribution. In Western Europe (the UK), adults are the most common on birds in the winter months, while the occurrence of larvae and nymphs was not sharply separated and occurred at any time between March and October [24]. These shifts are probably attributable to differences in the life cycle of *I. frontalis* under oceanic and continental climate in Europe.

Another ornithophilic tick species collected in this study is *I. arboricola*, which was previously found only a few times in Hungary [11, 25, 26]. Interestingly, so far nothing has been established about its seasonality in Hungary, but according to a foreign publication, it is most common before breeding and in the autumn and winter periods, but it can be found on both chicks and adults during the breeding season [27]. During this study, *I. arboricola* was collected on two occasions at the same ringing location, and both days were in December.

*Ixodes festai* was also found twice during this study, on Savi’s Warbler (*L. luscinioides*) and on Dunnock (*P. modularis*), in both cases in the spring, on migrating birds. This tick is a less-known species with a southern distribution, which, according to our current knowledge, does not breed in Central Europe or Hungary, but is only brought by birds coming from the south, from the Mediterranean region in the spring [7]. No one has previously reported this tick species from Savi’s Warbler (*L. luscinioides*) in Europe [12].

The species *I. lividus* was also collected four times from Sand Martin (*Riparia riparia*). This is a host-specific tick species; so far, it has only been described from Sand Martin (*R. riparia*) and once from Western House Martin (*Delichon urbicum*) [28]. It shows a strong seasonality, so the females that were collected in this study also occur in the summer on nestlings and first-year birds [29]. The hosts in this study represented the latter.

*Dermacentor reticulatus* has so far rarely been collected from birds in Europe [10, 12]. For the first time, it was found on a nymph from a Meadow Pipit (*Anthus pratensis*) [30] and for the second time from a larva from a European Robin (*E. rubecula*) [19]. On the other hand, during our investigation, a female was found on a Great Egret (*A. alba*) at a heron colony and for the second time on a Sedge Warbler (*A. schoenobaenus*) in northwestern Hungary. Therefore, this tick species was collected for the first time from both bird species in Europe [12]. In addition, no species of ticks have been previously reported from Great Egrets (*A. alba*) in Europe [12].

This is the first study which demonstrated differences in the feeding location of ticks on birds according to the habitat characteristics of the latter. The most common place of tick attachment was in the throat region (60%), especially in reedbed habitats. Contrarily, in a study carried out at the Baltic coastline, 75.0% of ticks occurred in the corner of the beak, 14.6% around the eyes, and only 4.4% on the throat, 4.4% in the ears, 1.1% on the back or the top of the head and 0.5% on the inside of the corner of the beak [19]. The most plausible explanation for the above differences between this and the Baltic study is that in the latter reed-associated bird species were underrepresented [19]. Since the predilection site of throat region for tick location was shown here to be associated with reed habitat, the likely reason for this phenomenon is bill wiping [31], during which the throat will come into contact with the reed stem on which questing ticks are situated.

## Conclusions

In conclusion﻿, aquatic birds appear to be less important in tick dispersal than songbirds. However, newly revealed tick-host associations in this category attest to their hitherto neglected contribution, including the occurrence of *I. ricinus* on Greylag Goose (*A. anser*) and *D. reticulatus* on Great Egret (*A. alba*). For the first time to our knowledge, ticks were collected in Europe from two species of predatory birds as well as from Little Bittern (*I. minutus*). Bird species typically inhabiting reedbeds were most frequently infested with *H. concinna*, and most ticks localized at their throat, as opposed to forest-dwelling avian hosts, on which *I. ricinus* predominated and ticks were more evenly distributed on various body parts. Thus, the results suggest that the habitat type will influence not only on the species composition but also the feeding location of ticks on birds.

### Supplementary Information

**Additional file 1.** List of encoded, Latin and English names of bird species that were found tick-infested in this study.

**Additional file 2.** Tick-infested birds according to the date of capture.

## Data Availability

All relevant data are included in the manuscript and its appendices.
