# Supplementary material for: Investigations of the tick burden on passeriform, water-associated and predatory birds reveal new tick–host associations and habitat-related factors of tick infestation
Source: Parasit Vectors. 2024 Mar 18;17:144. doi: 10.1186/s13071-024-06229-1 (PMC10949810; doi:10.1186/s13071-024-06229-1)
Supplement: Supplementary file 1 — Additional file 1. List of encoded, Latin and English names of bird species that were found tick-infested in this study. [file 13071_2024_6229_MOESM1_ESM.pdf]

Additional file 1. List of encoded, Latin and English names of bird species found tick-infested in this study.

HURING-code		Latin name	English name
1	LANCOL	*Lanius collurio*	Red-backed Shrike
2	PARCAE	*Cyanistes caeruleus*	Eurasian Blue Tit
3	PARMAJ	*Parus major*	Great Tit
4	PANBIA	*Panurus biarmicus*	Bearded Reedling
5	RIPRIP	*Riparia riparia*	Sand Martin
6	AEGCAU	*Aegithalos caudatus*	Long-tailed Tit
7	PHYCOL	*Phylloscopus collybita*	Common Chiffchaff
8	ACRARU	*Acrocephalus arundinaceus*	Great Reed Warbler
9	ACRMEL	*Acrocephalus melanopogon*	Moustached Warbler
10	ACRSCH	*Acrocephalus schoenobaenus*	Sedge Warbler
11	ACRSCI	*Acrocephalus scirpaceus*	Eurasian Reed Warbler
12	ACRRIS	*Acrocephalus palustris*	Marsh Warbler
13	LOCLUS	*Locustella luscinioides*	Savi's Warbler
14	LOCNAE	*Locustella naevia*	Common Grasshopper Warbler
15	SYLATR	*Sylvia atricapilla*	Eurasian Blackcap
16	SYLCOM	*Sylvia communis*	Common Whitethroat
17	REGIGN	*Regulus ignicapilla*	Common Firecrest
18	CERFAM	*Certhia familiaris*	Eurasian Treecreeper
19	CERBRA	*Certhia brachydactyla*	Short-toed Treecreeper
20	STUVUL	*Sturnus vulgaris*	Common Starling
21	TURMER	*Turdus merula*	Eurasian Blackbird
22	TURPIL	*Turdus pilaris*	Fieldfare
23	TURPHI	*Turdus philomelos*	Song Thrush
24	ERIRUB	*Erithacus rubecula*	European Robin
25	LUSSVE	*Luscinia svecica*	Bluethroat
26	LUSLUS	*Luscinia luscinia*	Thrush Nightingale
27	LUSMEG	*Luscinia megarhynchos*	Common Nightingale
28	PASMON	*Passer montanus*	Eurasian Tree Sparrow
29	PRUMOD	*Prunella modularis*	Dunnock
30	COCCOC	*Coccothraustes coccothraustes*	Hawfinch
31	CARCHL	*Chloris chloris*	European Greenfinch
32	EMBSCH	*Emberiza schoeniclus*	Common Reed Bunting
33	FALTIN	*Falco tinnunculus*	Common Kestrel
34	PERAPI	*Pernis apivorus*	European Honey Buzzard
35	AQUHEL	*Aquila heliaca*	Eastern Imperial Eagle
36	CIRAER	*Circus aeroginosus*	Western Marsh Harrier
37	IXOMIN	*Ixobrychus minutus*	Little Bittern
38	EGRALB	*Ardea alba*	Great Egret
39	ANSANS	*Anser anser*	Greylag Goose
40	COTCOT	*Coturnix coturnix*	Common Quail
