# Supplementary material for: Investigations of the tick burden on passeriform, water-associated and predatory birds reveal new tick–host associations and habitat-related factors of tick infestation
Source: Parasit Vectors. 2024 Mar 18;17:144. doi: 10.1186/s13071-024-06229-1 (PMC10949810; doi:10.1186/s13071-024-06229-1)
Supplement: Supplementary file 2 — Additional file 2. Tick-infested birds according to the date of capture. [file 13071_2024_6229_MOESM2_ESM.pdf]

Additional file 2. Tick-infested birds according to the date of capture.

	03.2021	04.2021	05.2021	06.2021	07.2021	08.2021	09.2021	10.2021	11.2021	12.2021	01.2022	02.2022	03.2022	04.2022	05.2022	06.2022	07.2022	08.2022	09.2022	10.2022	11.2022	12.2022	01.2023	02.2023	03.2023	04.2023	05.2023	06.2023	07.2023	08.2023	*Total*
**LANCOL**	-	-	-	-	-	-	-	-	-	-	-	-	-	-	-	-	-	-	-	-	-	-	-	-	-	-	-	-	-	1	1
**PARCAE**	-	-	-	-	-	-	-	-	-	-	-	-	-	-	-	-	-	-	-	-	-	2	-	-	-	-	-	-	-	-	2
**PARMAJ**	-	1	-	-	-	-	-	-	-	-	-	-	-	-	19	3	-	-	-	-	-	27	-	-	1	-	-	-	6	-	57
**PANBIA**	-	-	-	-	-	-	-	-	-	-	-	-	-	-	-	-	-	-	-	-	-	-	-	-	2	3	-	-	1	-	6
**RIRRIP**	-	-	-	-	-	-	-	-	-	-	-	-	-	-	-	2	-	-	-	-	-	-	-	-	-	-	-	2	-	-	4
**AEGCAU**	-	-	-	-	-	-	-	-	-	-	-	-	-	-	-	-	-	-	-	-	-	-	-	-	1	-	-	-	-	-	1
**PHYCOL**	-	-	-	-	1	-	-	-	-	-	-	-	-	-	-	-	-	-	-	-	-	-	-	-	1	-	-	-	-	-	2
**ACRARU**	-	1	-	-	1	-	-	-	-	-	-	-	-	-	-	-	3	5	-	-	-	-	-	-	-	-	-	-	5	1	16
**ACRMEL**	-	-	-	-	1	1	-	-	-	-	-	-	-	-	-	-	1	-	-	-	-	-	-	-	1	1	-	-	3	-	8
**ACRSCH**	-	-	-	-	7	1	-	-	-	-	-	-	-	-	-	2	2	7	1	-	-	-	-	-	1	4	-	1	17	-	43
**ACRSCI**	-	2	-	-	4	2	1	-	-	-	-	-	-	1	1	-	1	11	1	-	-	-	-	-	-	7	-	-	18	1	50
**ACRRIS**	-	-	-	-	1	-	-	-	-	-	-	-	-	-	-	-	6	2	-	-	-	-	-	-	-	-	-	-	8	1	18
**LOCLUS**	-	1	-	-	4	-	-	-	-	-	-	-	-	1	-	2	11	4	1	-	-	-	-	-	-	2	-	7	16	1	50
**LOCNAE**	-	-	-	-	-	-	-	-	-	-	-	-	-	-	-	-	-	-	-	-	-	-	-	-	-	-	-	-	-	1	1
**SYLATR**	-	-	-	-	-	-	1	-	-	-	-	-	-	-	-	-	3	-	-	-	-	-	-	-	-	-	-	-	2	1	7
**SYLCOM**	-	-	-	-	-	-	-	-	-	-	-	-	-	-	-	-	-	1	-	-	-	-	-	-	-	-	-	-	-	1	2
**REGIGN**	-	-	-	-	-	-	-	-	-	-	-	-	-	-	-	-	-	-	-	-	-	-	-	-	1	-	-	-	-	-	1
**CERFAM**	-	-	-	-	-	-	-	-	-	-	-	-	-	-	-	-	-	-	-	-	-	-	-	-	-	-	-	-	-	1	1
**CERBRA**	-	-	-	-	-	-	-	-	-	-	-	-	-	-	-	-	1	-	-	-	-	-	-	-	-	-	-	-	-	-	1
**STUVUL**	-	-	-	-	-	-	-	-	-	-	-	-	-	-	-	-	-	-	-	-	-	-	-	-	-	2	-	-	-	-	2
**TURMER**	-	-	-	-	-	1	-	-	1	-	-	-	-	-	-	-	1	1	-	-	-	-	-	-	6	-	-	2	4	-	16
**TURPIL**	-	-	-	-	-	-	-	-	-	-	-	-	-	-	-	-	-	-	-	-	-	-	1	-	-	-	-	-	-	-	1
**TURPHI**	-	-	-	-	-	-	-	-	-	-	-	-	-	-	-	-	1	-	-	-	-	-	-	-	-	-	-	-	-	1	2
**ERIRUB**	-	-	-	-	-	-	-	1	-	-	-	-	-	-	-	-	-	-	1	-	-	-	-	-	6	-	-	-	1	-	9
**LUSSVE**	-	-	-	-	-	-	-	-	-	-	-	-	-	-	-	-	-	-	-	-	-	-	-	-	-	-	-	-	1	-	1
**LUSLUS**	-	-	-	-	-	-	-	-	-	-	-	-	-	-	-	-	-	1	-	-	-	-	-	-	-	-	-	-	-	-	1
**LUSMEG**	-	-	-	-	-	-	-	-	-	-	-	-	-	-	-	-	-	-	-	-	-	-	-	-	-	-	-	-	5	4	9
**PASMON**	-	-	-	-	-	-	-	-	-	-	-	-	-	-	-	-	1	-	-	-	-	-	1	-	1	-	-	-	-	-	3
**PRUMOD**	-	-	-	-	-	-	-	-	-	-	-	-	-	-	-	-	-	-	-	-	-	-	-	-	17	-	-	-	-	-	17
**COCCOC**	-	-	-	-	-	-	-	-	-	-	-	-	-	-	-	-	-	-	-	-	-	-	-	-	-	-	-	2	-	-	2
**CARCHL**	-	-	-	-	-	-	-	-	-	-	-	-	-	-	-	-	-	-	-	-	-	-	-	-	2	-	-	-	-	-	2
**EMBSCH**	-	-	-	-	1	-	-	-	-	-	-	-	-	-	-	1	-	-	-	1	-	-	-	-	-	-	-	-	3	-	6
**FALTIN**	-	-	-	-	-	-	-	-	-	-	-	-	-	-	-	1	-	-	-	-	-	-	-	-	-	-	-	-	-	-	1
**PERAPI**	-	-	-	-	-	-	-	-	-	-	-	-	-	-	-	-	-	-	-	-	-	-	-	-	-	-	-	-	1	-	1
**AQUHEL**	-	-	-	-	-	-	-	-	-	-	-	-	-	-	-	-	-	-	-	-	-	-	-	-	-	-	-	-	1	-	1
**CIRAER**	-	-	-	-	-	-	-	-	-	-	-	-	-	-	-	1	-	-	-	-	-	-	-	-	-	-	-	-	-	-	1
**IXOMIN**	-	-	-	-	-	-	-	-	-	-	-	-	-	-	-	-	-	-	-	-	-	-	-	-	-	-	-	-	1	-	1
**EGRALB**	-	-	-	1	-	-	-	-	-	-	-	-	-	-	-	-	-	-	-	-	-	-	-	-	-	-	-	-	-	-	1
**ANSANS**	-	-	-	-	-	-	-	-	-	-	-	-	-	-	-	-	-	-	-	-	-	-	-	-	-	3	-	-	-	-	3
**COTCOT**	-	-	-	-	-	-	-	-	-	-	-	-	-	-	-	-	1	-	-	-	-	-	-	-	-	-	-	-	-	-	1
	-	5	-	1	20	5	2	1	1	-	-	-	-	2	20	12	32	32	4	1	-	29	2	-	40	22	-	14	93	14	352
